# Performance of an Electrothermal MEMS Cantilever Resonator with Fano-Resonance Annoyance under Cigarette Smoke Exposure

**DOI:** 10.3390/s21124088

**Published:** 2021-06-14

**Authors:** Andi Setiono, Michael Fahrbach, Alexander Deutschinger, Ernest J. Fantner, Christian H. Schwalb, Iqbal Syamsu, Hutomo Suryo Wasisto, Erwin Peiner

**Affiliations:** 1 Institute of Semiconductor Technology (IHT), Laboratory of Emerging Nanometrology (LENA), Technische Universität Braunschweig, 38106 Braunschweig, Germany; m.fahrbach@tu-braunschweig.de (M.F.); i.syamsu@tu-braunschweig.de (I.S.); h.wasisto@tu-braunschweig.de (H.S.W.); e.peiner@tu-braunschweig.de (E.P.); 2 Research Center for Physics, Indonesian Institute of Sciences (LIPI), Tangerang Selatan 15314, Indonesia; 3 SCL-Sensor.Tech. Fabrication GmbH, 1220 Vienna, Austria; a.deutschinger@gmx.at (A.D.); ernest.fantner@sclsensortech.com (E.J.F.); 4 GETec Microscopy GmbH, 1220 Vienna, Austria; chris.schwalb@getec-afm.com; 5 Research Center for Electronics and Telecommunication, Indonesian Institute of Sciences (LIPI), Jl. Sangkuriang-Komplek LIPI Gedung 20, Bandung 40135, Indonesia

**Keywords:** electrothermal piezoresistive cantilever sensors, parasitic feedthrough subtraction, particle mass concentration measurement, cigarette smoke exposure

## Abstract

An electrothermal piezoresistive cantilever (EPC) sensor is a low-cost MEMS resonance sensor that provides self-actuating and self-sensing capabilities. In the platform, which is of MEMS-cantilever shape, the EPC sensor offers several advantages in terms of physical, chemical, and biological sensing, e.g., high sensitivity, low cost, simple procedure, and quick response. However, a crosstalk effect is generated by the coupling of parasitic elements from the actuation part to the sensing part. This study presents a parasitic feedthrough subtraction (PFS) method to mitigate a crosstalk effect in an electrothermal piezoresistive cantilever (EPC) resonance sensor. The PFS method is employed to identify a resonance phase that is, furthermore, deployed to a phase-locked loop (PLL)-based system to track and lock the resonance frequency of the EPC sensor under cigarette smoke exposure. The performance of the EPC sensor is further evaluated and compared to an AFM-microcantilever sensor and a commercial particle counter (DC1100-PRO). The particle mass–concentration measurement result generated from cigarette-smoke puffs shows a good agreement between these three detectors.

## 1. Introduction

Resonant sensors (resonators) are frequency output devices with a vibrating element based on a mechanical resonance frequency that can change/shift as a function of a physical parameter. Its frequency output can be digitally sensed without an analog to digital converter (ADC) device and, therefore, can be directly linked to digital signal processing electronics [1]. The measured quantity is detected by converting to the resonance frequency through a change in stress, mass, or form of the resonator. Research and development in micro-electro-mechanical systems (MEMS) has led to the realization of highly sensitive and selective microscale sensors [2], including MEMS resonator devices, which have several advantages such as high sensitivity, high accuracy and large dynamic range. Recently, MEMS resonators have become favorable devices for ubiquitously measuring concentrations of any undesired substances (e.g., airborne nanoparticles, toxic gases, and humidity) by monitoring their responses in resonance frequency *f*_0_ [3,4].

MEMS resonators featured with thermal actuation offer the advantages of low operating voltage and ease of on-chip integration [5,6]. Thermal actuation is expected to have fewer difficulties in integration compatibility with circuits for signal processing [7]. Some research studies dealing with MEMS-based resonance sensors have indicated that they employ the thermal actuator due to its large actuation force [8], simple fabrication [9], and flexibility in the choice of materials [10]. Furthermore, piezoresistive-based sensing is also typically used in MEMS sensors besides capacitive [11], piezoelectric [12] or optical transducers [13]. Piezoresistors are the simplest form of piezoresistive devices with several advantages: low cost, small size, low phase lag, and extensive dynamic range [14]. Besides, the main attractive features of piezoresistive sensors are their high sensitivity and self-sensing ability [15], which become indispensable for detecting low mass concentrations such as chemical, biochemical, and biological analytes.

This work addresses the enhanced parasitic feedthrough subtraction (PFS) strategy on electrothermal piezoresistive cantilever (EPC) sensors as a thermally actuated cantilever beam that uses piezoresistive material as its transducer. The EPC sensors considered here were designed and exclusively fabricated in-house using *n*-type silicon-based material by employing bulk micromachining technologies, as described in [16,17]. To examine the sensor performance under a change in mass due to particle deposition on the cantilever, operation under cigarette smoke exposure was conducted. With a relatively stable distribution of sizes ranging from 0.1 to 1.0 μm (peaked between 0.2 and 0.25 μm) [18,19], cigarette smoke particles can be considered as a model for air pollution, which refers to a fine fraction with a size of up to 2.5 µm (PM 2.5). Furthermore, for comparison, this measurement was accompanied by simultaneous exposure monitoring using an AFM (atomic force spectroscopy) microcantilever sensor and a commercial particle counter (DC1100-PRO, Dylos Corporation, Riverside, CA, USA).

## 2. Materials and Methods

### 2.1. Enhanced Parasitic Feedthrough Subtraction (PFS) Method

**Figure 1:**
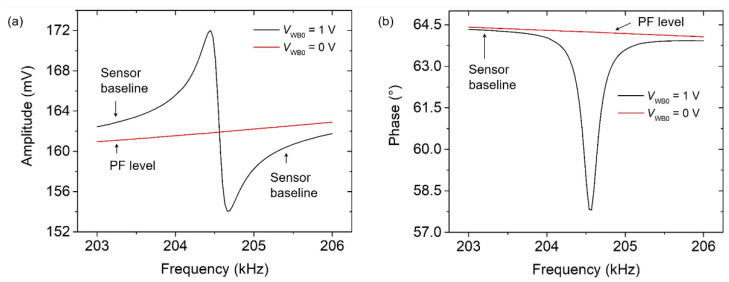
The level of parasitic feedthrough (PF) is recognized by setting the *V*_WB0_ = 0 V; therefore, the subtractor component can be determined by placing it close to the sensor baseline, either for (**a**) amplitude or (**b**) phase.

As described in [20], the alternating ohmic loss (*P*_ac_) causes temperature fluctuations (*T*_ac_) in the actuation part (HR). This further leads to nonlinear electromagnetic waves [21] that can interact with the silicon substrate and subsequently generate a parasitic element coupled to the WB output signal. Alternatively, a Fano resonance (asymmetric amplitude shape, black line in Figure 1a) has been investigated as a mixed state between a discrete state (Lorentzian line shape) and a constant continuum background [22], which subsequently represent the pure motional signal of the resonator and the parasitic feedthrough (PF), respectively. Therefore, regarding the appearance of Fano-shape resonances in the EPC sensor, temperature fluctuations (*T*_ac_) are most likely able to raise up a “continuum background” component that can subsequently couple to the WB output signal via the silicon substrate. Simultaneously, an asymmetric amplitude shape and reversing-phase response occurred as a result of the parasitic feedthrough (PF) coupling, as shown in Figure 1 (black line). To obtain the pure motional signal from the EPC sensor with optimized frequency response function (FRF), i.e., a symmetrical amplitude shape and monotonic phase response, a method of parasitic feedthrough subtraction (PFS) is further proposed and described in the following.

**Figure 2:**
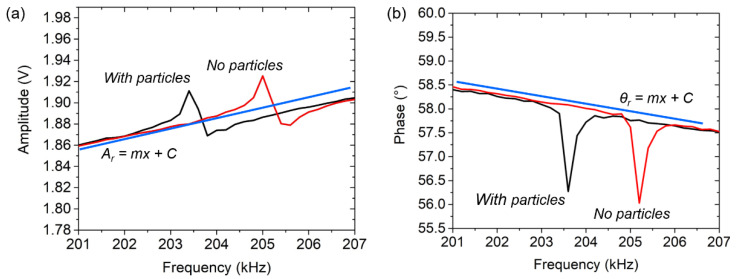
(**a**) An asymmetric amplitude response and (**b**) a reversing phase response is shifted due to particles added to the EPC sensor (PMMA, Polymethyl methacrylate from Sigma-Aldrich Inc., Taufkirchen, Germany) along a tilted axle line, revealing a frequency shift from *f*_0_ = ~205.2 kHz to *f*_0_’ = ~203.6 kHz. The PF component is estimated by the linear equation represented by the full blue line.

The PFS method comes with the objectives to eliminate the PF components by subtracting an external component from the output sensor, which is considered as a mimetic spectral representation or a “digital twin” of the PF component. To quantify or estimate the PF value, a WB input voltage of *V*_WB0_ = 0 V is selected [23], resulting in frequency-dependent PF levels following the sensor baselines either for the amplitude and phase (illustrated by the red lines in Figure 1). In this study, the PF subtraction was carried out digitally using a software application by implementing a differential approach, as shown in [20]. This was enhanced with self-adjusting of the subtraction component to anticipate the dynamic behavior of the sensor baseline. Figure 2a,b delineate the changes in the PF into different levels when the FRF of the EPC sensor shifted. In order to continuously maintain the optimized state of the sensor’s FRF, the subtraction components have to follow the sensor baseline. Therefore, there was a need for adaptive subtraction components that can automatically follow the dynamics of the sensor baseline. An equation-based subtraction component was subsequently proposed and actualized as a linear equation, shown as follows:

(1) $$A_{sub}=mf+C$$

(2) $$\theta_{sub}=mf+C$$

where *A_sub_*, *θ_sub_*, *m*, *f*, and *C* are subtraction amplitude, subtraction phase, slope or gradient, working frequency, and constant offset, respectively.

**Figure 3:**
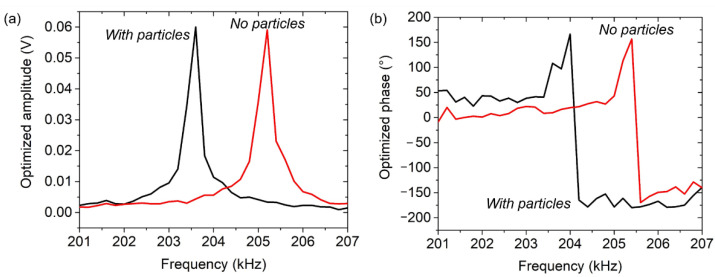
Application of equation-based parasitic feedthrough subtraction (PFS) method to an EPC with and without captured particles resulting in a horizontal shift of leveled (**a**) symmetrical amplitude shapes and (**b**) non-reversing phase responses.

As a result, Figure 3a,b evidently show that the optimized FRF can be obtained and maintained using the enhanced PFS in the frequency range of 201–207 kHz. The optimized response was captured to shift horizontally for both the optimized amplitude and the optimized phase. Although the optimized phase shown in Figure 3b does not display a clearly monotonic phase transition (due to a low resolution of data sampling (i.e., 200 Hz/step)), both of them (with and without added mass, i.e., black and red) present a similar transition curve between the phase lead (positive phase) and the phase lag (negative phase) across the resonance state, i.e., in the phase range of 150° to −150°. Nevertheless, the sensor’s baselines still depend on the stability of external variable conditions, such as temperature and humidity, which could impact on the invalid values of gradient *m* and offset *C*. Therefore, continuously updating the equation for every resonance measurement was necessary.

### 2.2. Measurement System for Resonant-Frequency Characterization and Tracking

**Figure 4:**
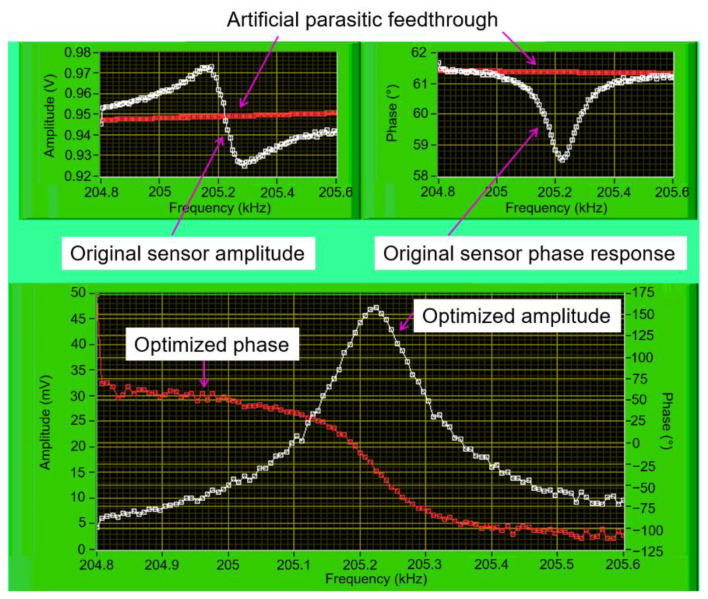
The LabVIEW front-panel of the frequency sweeper demonstrates an unoptimized FRF transformed to an optimized FRF, which is showed by a symmetrical amplitude shape (**white**) and a monotonic phase transition (**red**).

An application named “frequency sweeper” was used to evaluate the observed EPC sensor’s FRF. The principle of frequency sweeper is to generate a sequential frequency step around the EPC’s resonance state and subsequently use it to actuate the cantilever sensor. The FRF is defined as a magnitude of the output signals (amplitude response) and a phase difference between the actuation signal and the output signals (phase response). Regarding an unoptimized FRF (refers to asymmetric amplitude shape and reversing phase response) of the EPC sensor, we employed the optimizer equation, which was configured to a baseline-equation adaptive parasitic feedthrough subtraction (APFS). In this work, we used an advanced lock-in amplifier (MFLI, Zurich Instruments AG, Zurich, Switzerland) instrument and a compact FPGA-based system (Analog Discovery 2/AD2, Digilent, Pullman, WA, USA) as hardware systems. Furthermore, LabVIEW software was employed to perform the optimization procedures and access the resulting components of the demodulated signal to control the oscillator for optimizing the FRF. Figure 4 demonstrates that an optimized FRF (Lorentzian amplitude shape and monotonic phase response) can be constructed from an initially asymmetric amplitude shape and reversing-phase response. This optimized FRF subsequently became the bases to determine the resonance phase, which was then implemented in a PLL-based resonance tracking system.

**Figure 5:**
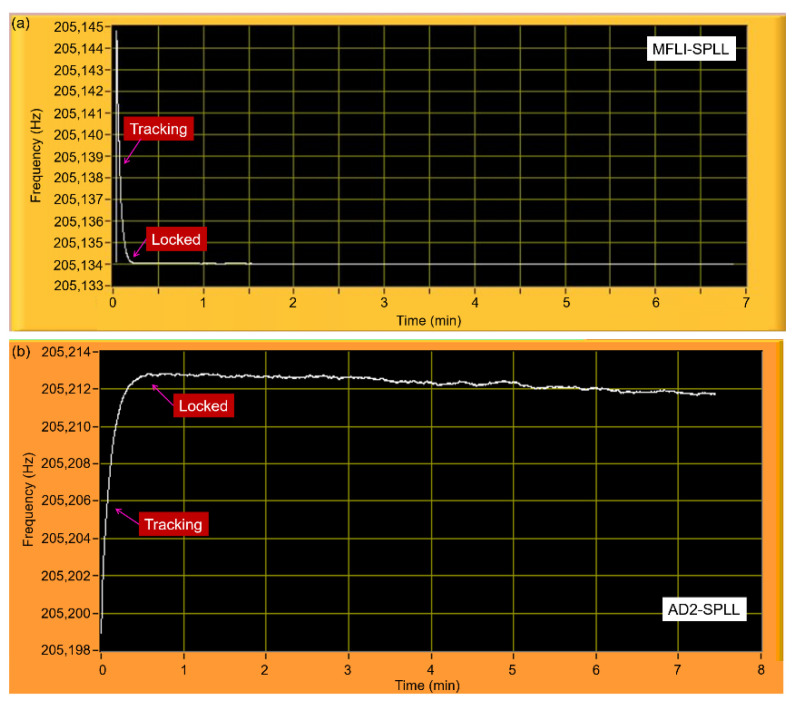
LabVIEW front-panel of (**a**) an MFLI-SPLL and (**b**) an AD2-SPLL demonstrating a resonance-locking procedure of an EPC.

For conducting the resonance tracking, (PLL)-based systems have been widely employed in resonator-based sensing schemes [17,24,25,26]. A PLL is a type of closed-loop control system that generates an output signal whose phase is related to the phase of the input signal. To realize it, a software phase-locked loop (SPLL) application was created as a counterpart of the frequency sweeper application. Basically, the developed PLL system works similarly to the frequency sweeper, i.e., the phase response is assessed from the demodulation system and subsequently fed into the optimizer equations. The yielded optimized phase was later compared to the opted resonance phase (set value), for which a-priori information was used from the optimized frequency responses yielded by the frequency sweeper application. Figure 5 demonstrates the LabVIEW front panel of the SPLL showing a locking process using a P-controller gain of 1 for the MFLI-SPLL (Figure 5a) exhibiting a tracking rate, a phase error, and a locked-frequency standard error of 1.25 Hz/s, 0.4 m° (milli degrees), and 0.3 mHz, respectively, whereas the AD2-SPLL shown in Figure 5b demonstrates a tracking rate of 0.5 Hz/s, a phase error of 17.6 m°, and a standard error of 1.6 mHz in the locked state. In the following, we further describe the implementation of the SPLL for resonance–frequency tracking in an environmental monitoring system.

## 3. Real-Time Cigarette Smoke Detection

**Figure 6:**
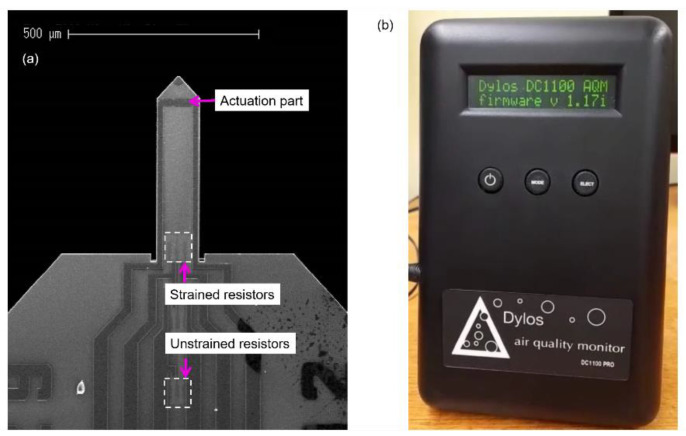
(**a**) SEM image of the AFM-MCS showing the thermal bimorph actuator at the cantilever’s free-end and four resistors configured in a half Wheatstone bridge configuration. (**b**) Photograph of the commercial optical-based particle counter DC1100-PRO.

After optimizing the FRF through the PFS strategy, the EPC sensor was subsequently assessed in a real-time measurement, i.e., tracking its resonance frequency under particle exposure. Measurement of cigarette smoke was opted to evaluate the performance of the EPC sensor and simultaneously compared with other two sensors, i.e., an AFM-MCS (atomic force microscopy microcantilever) and a commercial DC1100-PRO particle counter from Dylos Corporation (Riverside, CA, USA). It should be mentioned that all of these sensors are sensitive to arbitrary dust sources and do not specifically identify cigarette smoke. The AFM-MCS sensor is a commercial tip-less electrothermal cantilever sensor fabricated by SCL-Sensor.Tech. Fabrication GmbH (Vienna, Austria) and provided by GETec Microscopy GmbH (Vienna, Austria). Similarly to the EPC sensor, the AFM-MCS sensor is equipped with elements for electrothermal self-actuation and piezoresistive self-sensing. Here, it is in the form of an Al conductor–line bimorph actuator and a half Wheatstone bridge configuration (Figure 6a). Further information regarding the AFM-MCS sensor was described in [27]. Furthermore, the DC1100-PRO (Figure 6b) is an optical-based particle counter that is commercially available to measure particle number concentration (PNC) in the two size bins (>0.5 and >2.5 μm) [28]. The DC1100-PRO is a compact particle counter with dimensions of (18 cm × 12 cm × 8 cm) and a weight of 1.1 kg. This particle counter utilizes a photodetector which directly collects light scattered by particles entrained in a fluid traversing a beam of laser light [29].

**Figure 7:**
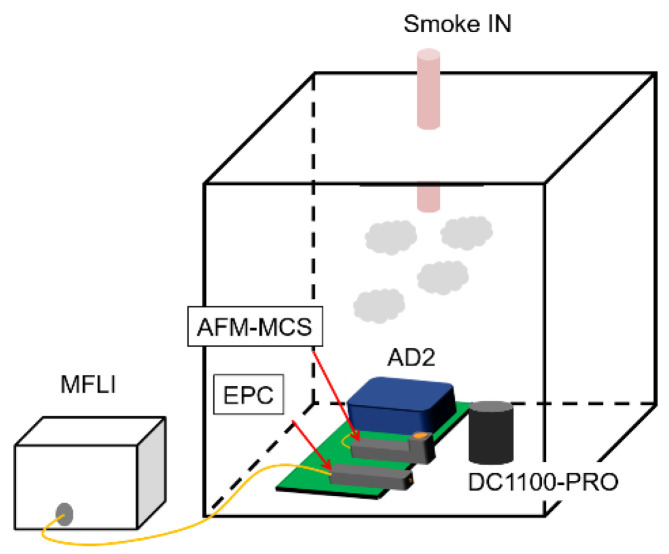
Schematic of a setup for cigarette smoke detection in a test chamber involving an EPC sensor, an AFM-MCS sensor, and a Dylos DC1100-PRO particle counter.

**Figure 8:**
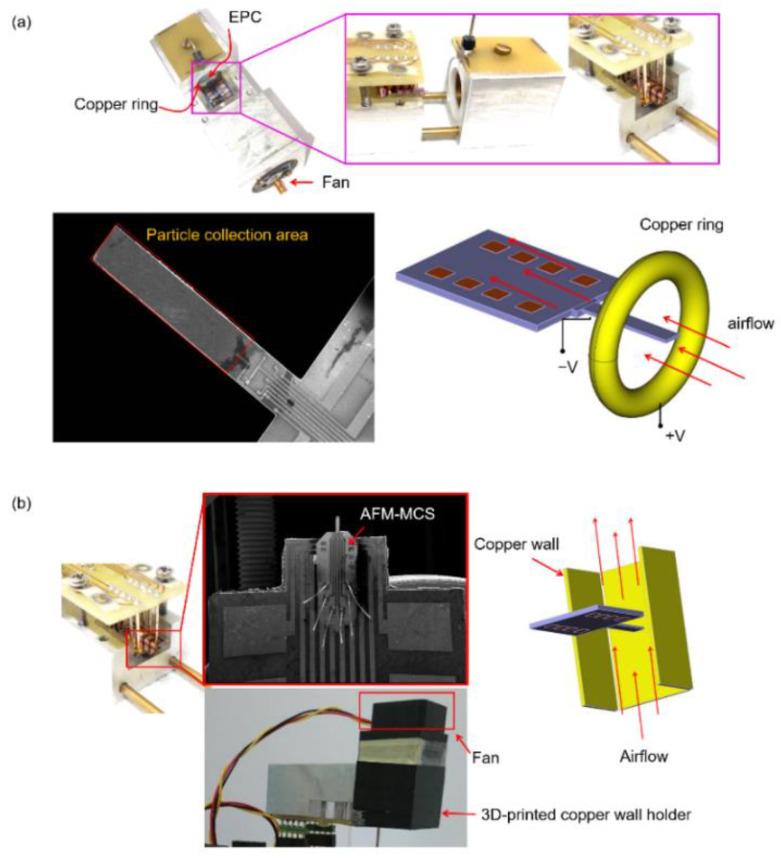
(**a**) Schematic of airflow direction in parallel to the cantilever beam axis driven by a small brushless fan (MF_10A03A by SEPA EUROPE GmbH, Eschbach, Germany) with airflow rate of 0.3 L/min. (**b**) Design construction of a setup based on an AFM-MCS sensor wired to the EPC substrate and surrounded by a 3D-printed copper wall holder. Airflow of 19.8 L/min is driven in a perpendicular direction to the axis of the AFM-MCS using axial DC fan (MF17080V2-10000-G99, Sunonwealth Electric Machine Industry Co., Ltd., Kaohsiung, Taiwan).

As delineated in Figure 7, these three sensors are placed in a box-shaped chamber made of plastic (0.086 m^3^) and simultaneously evaluated under exposure to the burned cigarettes. Figure 8 illustrates how airflow leads the smoke particles to enter the sampling zone of each sensor. On the cantilever sensors (EPC and AFM-MCS), the flowing smoke particles are collected by electrophoresis/dielectrophoretic attraction based on an electric field force conducted using positive and negative DC-high-voltages, which are connected to the metal electrode and cantilever beam’s electrode, respectively, as shown in [30,31,32]. However, the AFM-MCS is, essentially, not designed to be compatible with this electrophoresis/dielectrophoretic setup since it has no separate (special) electrode on its cantilever beam, which stands in contrast to the EPC sensor given in the form of a *p*-doped area (see Figure 8a). Therefore, to enable real-time particle-concentration tracking using the AFM-MCS sensor, particle collection was attempted by connecting only a positive high voltage to the surrounding electrode (copper wall, see Figure 8b) without employing a high negative voltage as a counterpart to the copper wall electrode. Considered to have a similar condition as the AFM-MCS sensor, the particle collection on the EPC sensor was also established with only the involvement of the high positive voltage, i.e., connected to the metal electrode (i.e., copper ring). On the other side, the cantilever beam electrode of the EPC sensor was left unconnected. Although the high negative voltage is not connected to the cantilever’s electrode, we can expect an electric field ending the trajectories of positive-charged particles to the cantilever beam. Furthermore, Brownian motion with a higher velocity [33] will induce a particle motion towards the cantilever surface where, eventually, van der Waals forces could lead to their being deposited [32,34].

Furthermore, the EPC sensor is actuated in an in-plane vibration mode and the airflow is directed in parallel to the cantilever axis at a low rate of 0.3 L/min, resulting in lower damping of the cantilever–beam oscillation. Instead, a particle-laden airflow at much higher rate (19.8 L/min) under vertical direction to the cantilever axis was applied to the AFM-MCS sensor, which can increase particle-trapping efficiency by impactation deposition. For the DC1100-PRO, the particles were sucked-in by an axial fan at low-pressure airflow (1.7 L/min) and subsequently passed through a laser beam on a small channel above the detector. When passing through the channel, the particles scatter a portion of the laser-light intensity onto the detector in which it is acquired. Larger particles have a larger scattering cross-section for the light compared to the small-particle fraction. Furthermore, to monitor the ambient conditions inside the chamber, a P5185 data logger from PeakTech is utilized, equipped with a K-type temperature sensor and a resistive-based hygrometer for humidity sensing.

**Figure 9:**
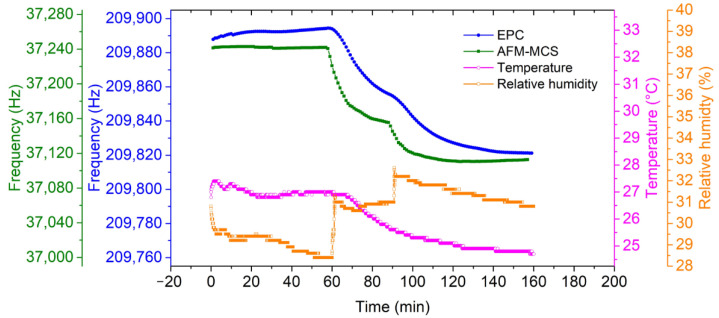
Resonance tracking of the EPC sensor demonstrated by the EPC sensor (blue full circle) and the AFM-MCS sensor (green full square) under ambient conditions (T = 25.9 ± 0.96 °C; rH = 30.5% ± 1.11%).

As mentioned above in Section 2.2, both the developed MFLI-SPLL and AD2-SPLL are employed to measure and track the resonance frequency of the EPC and the AFM-MCS sensor, respectively. The tracked resonance frequency of the EPC and AFM-MSC sensors during cigarette-smoke exposure, together with the measured relative humidity and temperature inside a closed box, is presented in Figure 9. In this measurement, a sequence of two-times cigarette-smoke puffs at 60 and 85 min are blown into the chamber, resulting in total resonance–frequency shifts of Δ*f*_0_EPC_ = ~−72 Hz and Δ*f*_0_AFM-MCS_ = ~−128 Hz. Obviously, the AFM-MCS sensor shows a larger resonance–frequency shift (Δ*f*_0_) than the EPC sensor, indicating that the AFM-MCS sensor has better sensitivity. The higher mass sensitivity (due to its mass [35,36,37]) is not completely canceled by the lower particle collection efficiency, which is expected due to the smaller surface area [34].

**Figure 10:**
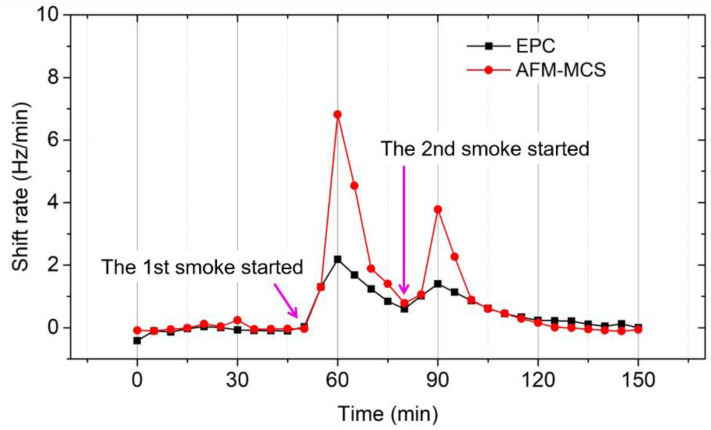
Frequency-shift rate at a 5-min sampling of cigarette smoke measured by EPC (full black square) and AFM-MCS (full red circle) sensors.

Moreover, as shown in Figure 10, time-coinciding peaks of the frequency-shift rates (for both sensors calculated over 5 min sampling time) were clearly demonstrated, indicating the appearance and removal (by deposition on the box walls) of the cigarette smoke. Additionally, average fluctuations of temperature and humidity within the chamber are observed to be ~0.96 °C and ~1.11%, respectively. Such fluctuations are expected for a chamber without an air-conditioning system. Referring to our result in [38], a change in relative humidity (RH) of 1% leads to a frequency shift (Δ*f*) of ~0.7 Hz on a bare EPC sensor. Hence, the increase in RH at 60 and 90 min can be estimated to have a contribution of only 3.1% to 4.4% to the measured Δ*f*_EPC_ under cigarette smoke exposure. Furthermore, Wasisto et.al [39] identified the temperature coefficient of the resonant frequency of a silicon cantilever of α_f_ = 28.6 ppm/°C. Accordingly, the decreasing temperature of Δ*T* ~2 °C in this work (see open circle line in Figure 9), according to Δ*f/f*_0_ = *α*_f_ × Δ*T*, led to a resonance shift of ~12 Hz. The corresponding influence on the frequency shift of ~73 Hz due to the attached cigarette smoke is about 16%. Therefore, smoke generation may be related to ambient temperature and humidity change (*T* and RH), but their interfering contribution to the measured Δ*f*_EPC_ can be neglected.

**Figure 11:**
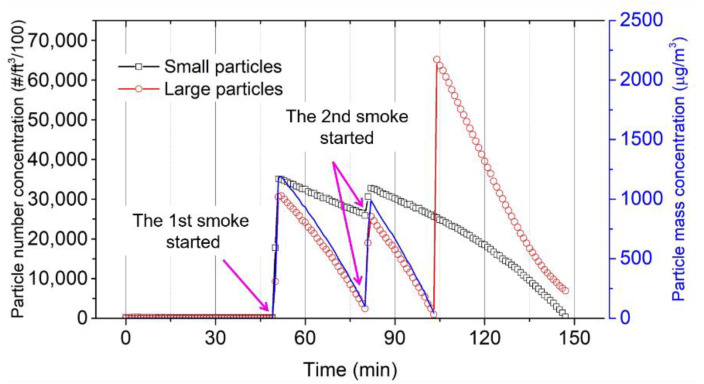
Small (open black square) and large (open red circle) particle number concentration and total mass concentration (full blue line) over time measured by the Dylos DC1100-PRO. The latter showed unreasonable mass concentrations at ~104 min, which can be assigned to a “rolled-over” response caused by excessive particle exposition.

Furthermore, the particle number concentration measured by the DC1100-PRO is demonstrated in Figure 11. The DC1100-PRO displays the concentration of particles in air assigned to two size bins, i.e., a small particle bin and a large particle bin, in multiples of 0.01 cubic foot of air. The small size bins are intended to collect particle sizes greater than 0.5 μm, while the particle sizes greater than 2.5 μm are assigned to the large size bin. Within a sequence of two-times cigarette smoke exposure, the DC1100-PRO shows the highest particle number concentrations of small and large particles, of ~3.3 × 106 #/ft^3^ and ~3.0 × 10^6^ #/ft^3^ particles, respectively. Nevertheless, there is an anomaly at around 100 min that shows a sudden increase to huge concentrations for the large-particle fraction. This is supposedly caused by an over-saturating of the sensor in which the numbers can “roll-over” due to a limitation of the analog to digital converter (ADC) to 16-bit variables (65,535 maximum value). This phenomenon can be expected due to the large amounts of cigarette-smoke particles sucked through the air channel inside the DC1100-PRO. To calculate the particle mass concentration, we used [40]:

(3) $$C_{m}=\rho \frac{\pi }{6}\left(N_{s}d_{s}^{3}+N_{l}d_{l}^{3}\right)\times 3531.5$$

where *N_s_* and *N_l_* are the number concentrations of small particles and large particles (in #/0.01 ft^3^), respectively. Furthermore, *d* is the diameter of the particles in meters, and *ρ* is the average density of particles in μg/m^3^. Since there are only two bins featured in the DC1100-PRO, according to work in [41], we established that *d_s_* = 0.44 μm and *d_l_* = 2.60 μm are attributed to small and large particles, respectively. Regarding the particle density, cigarette smoke particles are considered to have a spherical morphology and an average effective particle density of 1180 ± 113 kg/m^3^ [40]. The constant factor of 3531.5 is employed to convert the concentration unit from #/0.01 ft^3^ to #/m^3^. As exhibited by the full blue line, the mass concentration measured by the DC100-PRO is dominated by the large particle fraction due to their larger mass.

**Figure 12:**
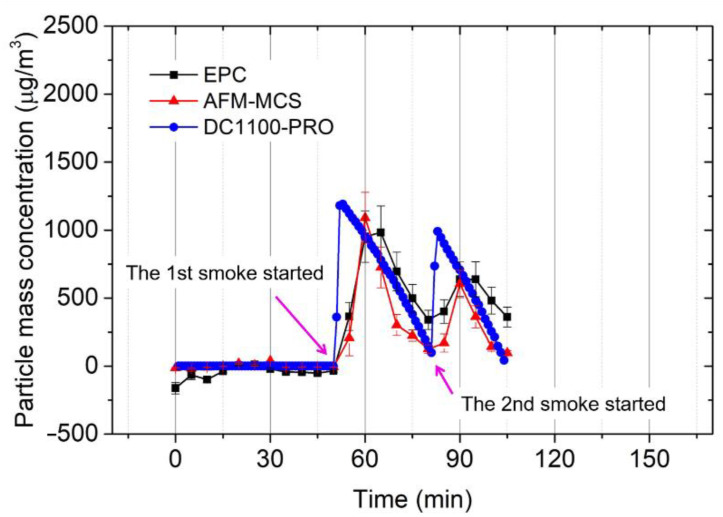
Mass concentration of the cigarette soot particles measured by the EPC sensor (full black square) and the AFM-MCS sensor (full red triangle) in comparison with the Dylos DC1100-PRO (full blue circle).

To compare the examined sensors (EPC and AFM-MCS) with the DC1100-PRO, Figure 12 is presented. Calibration factors (CF) of 500 μg × min/(m^3^ × Hz) and 160 μg × min/(m^3^ × Hz) are employed to convert the frequency-shift rate of the EPC and the AFM-MCS sensor, respectively, to the mass concentration regime formulated as follows [17]:

(4) $$C_{m}=CF\times \frac{\Delta f_{0}}{t_{CT}}$$

where *C_m_* and *t_CT_* are nanoparticle-mass concentrations measured by the cantilever sensor and particle collecting time (sampling time), respectively.

As a first qualitative feature, the cantilever sensors apparently exhibit a slower response compared to the DC1100-PRO. This can be assigned to different sensing principles where the DC1100-PRO can detect the passing particle in the channel immediately. Simultaneously, as a collection-based device, the cantilever sensor requires a minimum amount of particles deposited on the mass-sensing element, which leads to a time delay. Furthermore, it should be remembered that with the absence of the counter electrode of the collecting voltage on the cantilever beam, the efficiency of the positively charged particle collection might be less. Norman and Keith [42] reported that 47.3% of cigarette smoke particles in their study had few charges under an imposed electrical field, while 1.2% were found to have considerably larger charge numbers, and the remaining 44.6% of the particles were uncharged. With almost 50% of smoke particles being charged (albeit most of them in small amounts), we expect that these particles can be successfully attracted towards the cantilever. Here, particle deposition, especially for the humid fractions of cigarette smoke, will be driven by van der Waals forces. As indicated in [40,43,44], water content and sticky components (such as tar) are contained in cigarette smoke. However, in general, either the EPC or the AFM-MCS can properly detect the appearance of the cigarette soot in the chamber. The EPC and the AFM-MCS sensors’ deviation (error bars) are averaged at ~54 and ~32 μg/m^3^, respectively. Simultaneously, a much lower noise floor of *σ*__EPC_ = ~0.007 Hz and *σ*__AFM-MCS_ = 0.014 Hz, resulting in limits of detection of LOD_EPC_ = 21 μg/m^3^ and LOD_AFM-MCS_ = 1.3 μg/m^3^, respectively, was found. The noise floor was achieved by averaging the standard error (SE) of the measured resonance frequency according to the sampling time. Here, we assign this low noise floor to the frequency tracking without connecting the cantilever electrode to the high negative potential, as would be necessary for the Cantor device [3,17]. However, as a drawback, particle-collecting efficiency was considerably lower.

Finally, owing to its much smaller surface area, the particle collection in the AFM-MCS is expected to be much less efficient than in the EPC sensor. Furthermore, with its higher pressure drop and particle velocity, small particles in the range of 0.5 to 1 μm, or below 0.5 μm, with much lower inertia than the large-size fraction, may be attracted in a much smaller amount to the AFM-MCS cantilever. This may explain why the EPC sensor can still detect a certain amount of mass concentration when the signals of the DC100-PRO and the AFM-MCS have decreased towards zero. Figure 11 confirms that small particles may still be recorded in sufficiently large numbers even if the large-size fraction is already low. Hence, in conclusion, with the existing limitations, the AFM-MCS and the DC100-PRO provide good performance at large particle mass concentrations. At the same time, the EPC sensor can also detect mass concentrations of small particles.

## 4. Conclusions

A parasitic feedthrough subtraction (PFS) method was described, which essentially uses the parasitized signal read-out from an electrothermal piezoresistive cantilever (EPC) sensor and modifies it with an external component. A digital twin of the parasitic signal is created, which is then subtracted from the sensor signal. A programmable subtraction component was developed in LabVIEW for extracting the parasitic elements from the sensor output signal, from which it was subsequently internally subtracted. As it is feasible for any parasitized EPC sensor, an optimized FRF could thus be effectively realized. This software-based PFS method was then implemented in frequency-sweeper and software phase-locked loop (SPLL) applications to conduct a frequency-resolved resonance state characterization and real-time resonance–frequency tracking, respectively. Applications of the PFS method in airborne-particle concentration measurement using the EPC sensor showed strong performance and resulted in responses that were in good coincidence with a commercial optical particle counter. The EPC sensor was operated together with an atomic-force microscopy microcantilever sensor (AFM-MCS) and the commercial portable particle counter Dylos DC1100-PRO under cigarette smoke exposure up to ~1 mg/m^3^. In this range, the EPC and the AFM-MCS sensors show average deviations of ~50 and ~30 μg/m^3^, respectively, with respect to the DC1100-PRO.

## Data Availability

Not applicable.
